# Activity-based mindfulness: large-scale assessment of an online program on perceived stress and mindfulness

**DOI:** 10.3389/fpsyg.2024.1469316

**Published:** 2024-10-15

**Authors:** Eliane Timm, Yobina Melanie Ko, Theodor Hundhammer, Ilana Berlowitz, Ursula Wolf

**Affiliations:** ^1^Institute of Complementary and Integrative Medicine, Faculty of Medicine, University of Bern, Bern, Switzerland; ^2^Eurythmy4you, Nidau, Switzerland

**Keywords:** mindfulness-based intervention, mindfulness, stress, integrative medicine, online intervention, anthroposophic medicine, mind–body interventions, activity-based stress reduction

## Abstract

**Background and objective:**

Mindfulness has emerged as key construct in mental health over past decades. While current mindfulness-based interventions (MBIs) are usually rooted in Asian contemplative traditions, mindfulness practices can equally be found in other knowledge systems, including integrative medicine systems such as anthroposophic medicine (AM). The *Activity-Based Stress Release* (ABSR) program incorporates the latter as part of an 8-week-long online intervention combining mindfulness exercises, behavioral self-observation, and mindful movement practices derived from this integrative medicine frame. The program could offer additional means for cultivating mindfulness, thereby addressing the necessity for diverse approaches in conjunction with individual differences, diverse clinical demands, or restricted capacities to perform certain mindfulness practices. Using an observational repeated-measures design, the current study aimed to assess a large-scale online implementation of this program in terms of its feasibility, assessing perceived stress and mindfulness.

**Method:**

Individuals who enrolled in any of the 37 ABSR program iterations carried out during 2023 and agreed to participate in the study completed online surveys including validated stress and mindfulness scales at the beginning, middle, end, and follow up of the intervention. Linear-mixed models were used for data analysis.

**Results:**

A total of 830 individuals took part in the study, of which 53.5% filled in at least 2 surveys. In line with our expectation, mindfulness scores increased significantly over the course of the intervention, while stress scores decreased significantly in this timeframe. We further found differential effects of self-practice frequency and duration on the outcomes.

**Conclusion:**

This study provides a first indication of stress reduction in conjunction with the online implementation of this novel MBI. The work further suggests that this AM-based intervention indeed targets mindfulness, as do other MBIs, and that it is adaptable to an online format. However, given the observational single-arm design, controlled studies will be necessary to confirm these results. Nonetheless, the study adds a novel contribution to existent MBIs, which is significant in view of the need for diverse approaches to meet the heterogeneity of individual predispositions and clinical requirements. It remains to established by forthcoming research for which groups of individuals or clinical features this approach could be especially beneficial or less suitable.

## 1 Introduction

Mindfulness has emerged as key construct in mental health over past decades (Cullen, 2011; Galante et al., 2021a; Lee et al., 2021). Since the seminal work of Kabat-Zinn (1990) introducing the Buddhist mindfulness concept—a specific, nonjudgmental present-moment awareness – to Western health science, mindfulness-based interventions (MBIs) have exponentially increased and in some variations have become an integral part of so-called third-wave cognitive behavioral therapies (Khoury et al., 2015; Segal et al., 2018; Garland et al., 2012; Kabat-Zinn, 2003a; Hayes and Hofmann, 2021).

Kabat-Zinn’s original “Mindfulness-based Stress Reduction” (MBSR, Kabat-Zinn, 2003b) emphasized psychological stress as fundamental target variable based on its critical role in the chronic mobilization and dysregulation of the neurophysiological stress response, which in turn is associated with increased risk for non-communicable diseases and mental illnesses (Lagraauw et al., 2015; Vanitallie, 2002; Karami et al., 2023; Sinha and Jastreboff, 2013; Gold, 2015; Sapolsky, 2007; McEwen and Morrison, 2013). Subsequent adaptations of the MBSR have been focusing on specific mental health or somatic conditions (e.g., depression, chronic headache, chorioretinopathy), and continued to contribute to the accumulating evidence of MBIs’ clinical benefits (Kriakous et al., 2021; Yu et al., 2023; Goldsmith et al., 2023; Smithers-Sheedy et al., 2024; Özcan and Karapapak, 2024; Hoge et al., 2023; Fisher et al., 2023; Anheyer et al., 2019).

While the conceptual frame and practices of the original MBSR programs stem from Eastern and particularly Buddhist teachings (traditionally based particularly on the Satipaṭṭhāna Sutta, see Analayo, 2003), which indeed possess exceptional insight regarding mindfulness (“sati”) and other mental/psychological processes (for Buddhist psychology see the Abhidhamma; Bodhi, 2012), there is nothing inherently Buddhist about mindfulness itself, as Kabat-Zinn (2003a) and others (Meaden, 2024) pointed out. Rather, mindfulness should be understood as an innate human capacity, which arises spontaneously under certain circumstances and can be cultivated with various means.

Indeed, albeit under different names, many contemplative and traditional medicine systems of the world describe concepts akin to mindfulness and related practices, not limited to Eastern traditions like Yoga/Ayurveda (Salmon et al., 2009; Mamtani and Mamtani, 2005) or Traditional Chinese Medicine (e.g., Qi Gong, Tai Chi) (Fogaça et al., 2021; Atkins, 2018), but also extending to Indigenous knowledge systems, like the Australian Aboriginal concepts of “Dadirri” or “Ngarraanga Giinganay” (Ungunmerr, 2017; Lavrencic et al., 2021), the immediacy of experience principle of the Brazilian-Amazonian Pirahã, and related concepts by the Congolese Mbuti (Meaden, 2024). Indeed, also from an academic viewpoint, there is no clear consensus as to the defining features of mindfulness practices (Sedlmeier, 2023).

Mindfulness-related practices are also an important pillar of anthroposophic medicine (AM), a well-established integrative medicine framework that originally arose in the early nineteen-hundreds in Central Europe (Bartelme, 2020; Büssing et al., 2011) with some degree of Buddhist influence (Dahlin, 2009; Haas, 2017; Steiner, 1932; Steiner and Dietler, 2006). Today AM is integrated in many hospitals and clinics across Europe and over 60 countries around the world. The approach has originally developed from and fully includes modern conventional medicine and other associated clinical sciences, but extends these concepts with therapeutic approaches that focus on the person as a whole and employs a strongly patient-centered approach (Baars et al., 2017; Kloter et al., 2023). As an integrative and multimodal treatment system, AM thus combines methods from conventional biomedicine, psychotherapy/ counseling, and nursing, with complementary methods involving herbal medication, art and movement therapies, and massage techniques (Kienle et al., 2013), aiming to account for the integrity of human experience which consists of physical, psychological, social, as well as spiritual aspects. As is characteristic of complementary medicine systems, mental health is thus addressed as part of the integral therapeutic approach, rather than as a segregated discipline.

Within this system, the importance of mindfulness is articulated in a range of therapeutic approaches, including a mindful movement practice labeled eurythmy therapy, which involves movements linked to speech-sounds performed in a state of focused concentration to connect body movements with inner sensation (Kirchner-Bockholt, 1977; Berger et al., 2015), meditative practice, as well as specific task- or activity-based exercises (see section 2.2 for further details) derived from the AM treatise on approaches to counteract mental restlessness (“nervousness”; Steiner, 2009b, Steiner, 2009a, Kirchner-Bockholt, 1977, von Laue and von Laue, 2010).

The current study aimed to assess the online delivery of an MBI that draws from the latter AM concepts. Labeled *Activity-Based Stress Release* (ABSR) program, the intervention was based on the MBSR in structure and was originally developed as an in-person group therapy supporting psychiatric outpatients (Haas and Hundhammer, 2013). The in-person program has been described in a qualitative account (Haas and Hundhammer, 2013) and was evaluated in a small-scale pilot study based on a clinical sample of *N* = 20 patients (depression, anxiety disorders, or burnout diagnoses) with preliminary indications for improvements in calmness and serenity scales, heart rate variability, and in parasympathetic activity, although physiological parameters were assessed only in a small subsample (*n* = 4) (Kloter et al., 2023). The program represents a novel contribution from a well-established complementary medicine framework, which could offer additional means for cultivating mindfulness. This work thereby addresses the necessity for diverse MBIs in view of individual differences, diverse clinical demands, or restricted capacities to perform certain mindfulness practices. Disposing of multiple distinct approaches is of interest given that ‘one-size-fits-all’ approaches generally fall short of meeting the complexity and heterogeneity characteristically encountered in the mental health field. However, a large-scale assessment of the program has not yet been conducted, and the feasibility of its online implementation remains to be established.

Using an observational repeated-measures design, the objective of the current study was to assess the online implementation of the ABSR program in terms of intended effects and adaptability of the intervention to the online context (see types of feasibility; Bowen et al., 2009) based on a large-scale cross-cultural sample for feasibility testing. More specifically, using a general international sample, the study aimed to assess (a) stress and (b) mindfulness outcomes in conjunction with the online delivery of the ABSR program. By including mindfulness as an outcome we sought to test if this program indeed targets mindfulness, as is the case for other MBIs. We hypothesized that, compared to baseline, there will be a substantial (a) reduction in stress and (b) increase in mindfulness at the end of the intervention and at follow up.

## 2 Methods

### 2.1 Study design and setting

The study was conducted by the University of Bern’s Institute of Complementary and Integrative Medicine in collaboration with an accredited health provider specializing in the ABSR model.^1^ To assess the online implementation of the ABSR program (see section 2.2 for a detailed description of the intervention), we used a longitudinal repeated measures design with four assessment times to explore changes in stress (primary outcome) and mindfulness (secondary outcome). The four measurement times included baseline (t0), midpoint (t1), program completion (t2), and follow-up (t3).

Given full anonymity of participants at all stages of the research (fully encrypted survey via anonymous self-generated codes, no collection of IP- or E-Mail addresses, no collection of identifying participant data), no ethics/IRB approval was necessary according to the responsible Ethics Committee (Swiss Association of Research Ethics Committees) guidelines and the Federal Act on Research involving Human Beings (Human Research Act, 2011). Facilitators of the ABSR program did not have access to the survey data, which were collected by independent researchers (ET, YMK; University of Bern).

### 2.2 Intervention

The ABSR program consisted of an eight-week-long online intervention aimed to relieve stress via the cultivation of mindfulness using practices from AM. The intervention was based on weekly 90-min-long live online sessions, during which a trained facilitator introduced each of the weekly themes (8 modules in total, see Table 1) and taught participants the corresponding exercises. Table 1 shows the exercises per module, structured into mindful movement practice exercises (Eurythmy Therapy) and activity-based exercises. The latter label aims to articulate that these types of exercises, although performed with a contemplative attitude, require the active performance of defined tasks involving physical and/or mental operations. Participants were encouraged to practice the exercises over the course of the week (recommendation to practice on a daily basis, at least 15 min per exercise), and during the subsequent online session had the opportunity to discuss experiences from the preceding week, as well as address questions. Together with the closing session the program thus involved 9 online sessions (i.e., a total of 13.5 h of live sessions) and an individually varying amount of self-practice across 8 weeks. Audiovisual material and a forum for exchange outside the live sessions were available on the web portal, and in case participants missed a live session they could also access the recording there.

**Table 1:** ABSR modules, themes, and mindfulness exercises.

Module	Aspects of mental restlessness/stress^a^	Activity-based exercises	Eurythmy therapy
1	Forgetfulness due to inattention	Conscious misplacement of objects	A (breathing)
2	Nervousness and anxiety	Deliberate modification of handwriting	O (warming)
3	Self-doubt and worry	Reverse order thinking exercises	I (nourishing)
4/5	Restlessness and loss of control	Self-observation; changing habits	E (in−/excreting)
6	Dependencies	Non-reaction to small desires	Ei (maintaining)
7	Compulsions and indecision	Conscious decision-making exercises	Au (growing)
8	Rumination and obsessive thinking	Non-reaction to self−/criticism	U (generating)

a Originally termed “aspects of nervousness” in STEINER (2009b).

Data collection took place between September 2023 and March 2024. During this time period, the 8-weeks-long ABSR program was carried out a total of 37 times. Each of these were held by a certified ABSR facilitator, in groups of varying sizes (size being determined based on number of registrations, 2–264 registrations/group) and languages (English, German, Russian, Ukrainian, Slovenian, Dutch, Finnish, Chinese, and Spanish). All facilitators had undergone a standardized comprehensive ABSR certification training imparted by an accredited health provider (see footnote 1) prior to their involvement in this study. The training included four components, namely extensive self-experience with the ABSR practices and the program as a whole as a participant, attending a sequence of training lectures, completing a practicum in which trainees had to teach ABSR-related exercises in a group setting, as well as a final assessment by means of a written report or presentation. Certification was contingent upon successful completion of all four components. ABSR enrolment was fee-based, but rates were kept at the necessary minimum and subsidized spaces were available for those lacking the required means.

### 2.3 Participants and procedure

The online program and the study were advertised on the health provider’s website, in health magazines and newsletters, clinics also offering AM services, physicians’, psychiatrists’, and psychotherapists’ practices, as well as on social media. All individuals who enrolled in any of the 37 ABSR program iterations held between September and December 2023 were invited to participate in the study. They were thoroughly informed about the study and the voluntary nature of participation and provided implied informed consent by completing the survey (opt-in). Individuals who agreed to participate and completed at least one survey were included in the study. Those who were below 18 years of age or participated in more than one cycle of the program were excluded from the study. To assess the outcomes and descriptive items (see section 2.4 for a detailed description of measures) participants were given a survey link upon registration (up to 3 days before program start; t0), again 4 weeks later (t1) at the program’s midpoint, again another 4 weeks later (t2) at program completion, and again 8 weeks later (t3) at follow up (i.e., 16 weeks after baseline). Data collection ended when the last program cycle’s t3 measures were completed (March 2024).

### 2.4 Survey

The online survey was constructed by means of the *SoSci Survey* software (Leiner, 2023) and was made available in six languages (English, German, Chinese, Spanish, Russian, and Ukrainian). Aside from anonymized basic demographic information, it involved the following measures:

#### 2.4.1 Stress

The Perceived Stress Scale (PSS-10; Cohen et al., 1983, official validated translations from Mapi Research Trust 2022 © Copyright) is a 10-item instrument designed to assess experienced stress during the past month. Each item is rated on a five-point Likert scale from 1 (*never*) to 5 (*very often*), with higher scores indicating greater perceived stress (total sum scores: 0–40).

#### 2.4.2 Mindfulness

We employed the Mindful Attention Awareness Scale (MAAS; original English: Brown and Ryan, 2003, validated Chinese, German, Spanish, and Russian versions: Chen et al., 2012, Michalak et al., 2008, Barajas and Garra, 2014, Golubev, 2012), which is commonly used in clinical research to assess mindfulness (MacKillop and Anderson, 2007; Carlson and Brown, 2005). The instrument’s 15 items are rated on a six-point scale from 1 (*almost always*) to 6 (*almost never*), with higher MAAS scores corresponding to higher levels of mindfulness.

#### 2.4.3 Self-practice time and online participation frequency

To assess the frequency with which participants engaged in self-practice between sessions, they were asked how many days per week they had practiced the exercises in the weeks since the last assessment (possible answers: *0–1 days*, *2–3 days*, *4–5 days*, *6–7 days*) and, to assess the practice duration, how much time they had spent on the exercises per day (options: *not done*, *1–10 min*, *11–20 min*, *21–30 min*, *more than 30 min*).

### 2.5 Data analysis

All statistical analyses were performed using *R* version 4.4.0 (R Core Team, 2024). We used descriptive statistics to report sample characteristics and additional descriptive items. For all inferential analyses the significance level was set at *α* < 0.05. Surveys that were filled in outside the predefined time windows (i.e., less than 2 weeks apart from each other for t0-t2, more than 7 weeks between t0 and t1, or less than 4 weeks between t2 and t3) were excluded from the analysis. We opted for relatively broad time windows to avoid extensive data loss, but additionally performed all analysis also with a more narrow time window (at least 3 weeks and maximally 6 weeks), which however did not yield any different results.

All analyses were based on the data from all participants, non-completers included. We performed Linear Mixed-Effect Models (LMM) for each outcome variable in order to test whether there were significant changes in participants’ stress or mindfulness levels over time, assessing differences between the various measurement points. We opted for LMM because of the method’s suitability for analyzing repeated-measures data and for describing variations of the target variable across time, and importantly also due to the method’s capacity to calculate unbiased model estimates even in the face of extensive missing data, which is a notorious challenge in longitudinal studies in general, and particularly when conducted online (Krueger and Tian, 2004; Gabrio et al., 2022). For the LMM calculations we used the *R* packages *lme4* (Bates et al., 2015) and *nlme* (Pinheiro et al., 2023). All models were adjusted for age and sex, as well as survey language as a proxy for culture, as covariates.

Finally, we performed a series of one-way ANOVAs to test if the frequency and duration of self-practice had an impact on the outcomes. In other words, we tested if individuals who practiced for longer or more frequent intervals vs. those with shorter or less frequent self-practice showed significant differences in their stress or mindfulness levels at subsequent time points. We opted for one-way ANOVAs to test this due to the method’s capacity to compare differences between various group means (Mishra et al., 2019). The ANOVAs that involved practice frequency as predictor compared 4 groups (namely, the groups of individuals who practiced 0–1, 2–3, 4–5, or 6–7 days per week) whereas for the ANOVAs in which practice duration was the predictor, the comparison involved 5 groups (i.e., individuals who reported 0, 1–10, 11–20, 21–30, or >30 min of practice per reported practice day).

## 3 Results

### 3.1 Sample characteristics

**Figure 1:**
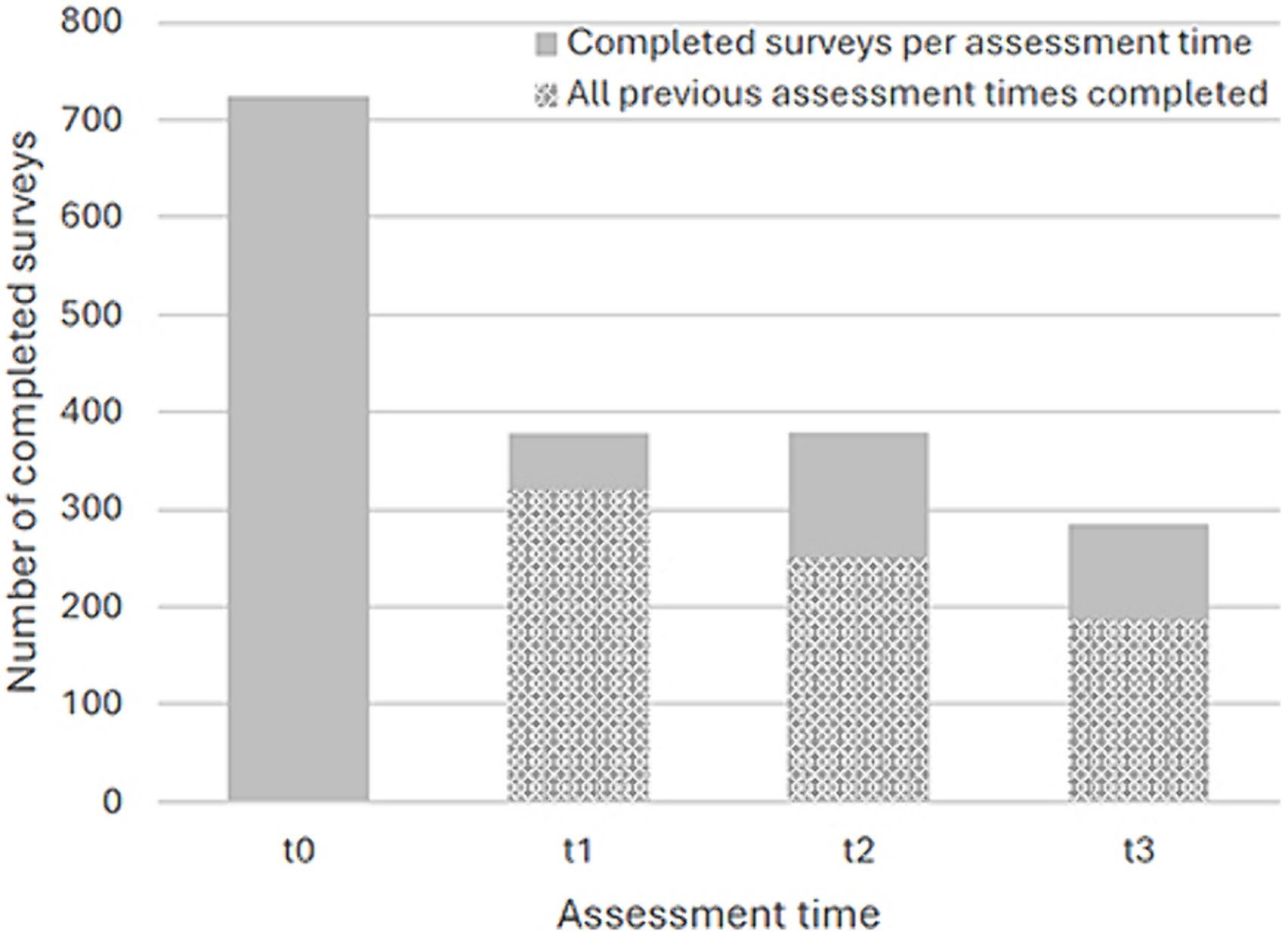
Number of completed surveys per assessment time.

Overall 1,155 individuals registered in the 37 implementations of the program (English-language implementations had *n* = 130 registrations, German: *n* = 259, Chinese: *n* = 264, Russian: *n* = 183, Ukrainian: *n* = 200, Spanish: *n* = 19, Finnish: *n* = 33, Dutch: *n* = 17, and Slovenian: *n* = 50) of whom 830 agreed to participate in the study and filled in the minimally required survey, as per inclusion criteria. Of the full sample (*N* = 830), 444 (53.5%) filled in at least two surveys and 186 (22.4%) filled in all four surveys (see Figure 1 for completed surveys per assessment time). Table 2 shows the full sample’s demographic characteristics and language in which the surveys were filled in. The majority of participants were middle aged, female, and of a European context. Table 3 shows the sample’s baseline levels of stress and mindfulness. The sample’s PSS-10 baseline score was indicative of moderate stress levels (Adamson et al., 2020) and above the norms for healthy adults (Cohen, 1988), whereas the baseline MAAS score was somewhat below normative general population samples (Carlson and Brown, 2005; Brown and Kasser, 2005).

**Table 2:** Sample characteristics.

		*n*	%
**Age distribution (years)**			
	18–31	32	3.9
	31–40	162	19.5
	41–50	263	31.7
	51–60	203	24.5
	61–70	132	15.9
	>70	32	3.9
	NA	6	0.7
Mean age (SD)		49.79 (11.72)	
**Gender**			
	Female	730	88
	Male	96	11.5
	Diverse	0	0
	NA	4	0.5
**Survey language**			
	English	177	21.3
	German	203	24.5
	Chinese	197	23.7
	Spanish	19	2.3
	Russian	149	18
	Ukrainian	85	10.2

NA, not available.

**Table 3:** Self-reported stress and mindfulness per assessment time.

	t0		t1		t2		t3	
	*n*	*M* (SD)	*n*	*M* (SD)	*n*	*M* (SD)	*n*	*M* (SD)
PSS-10	702	20.21 (6.00)	367	16.86 (5.58)	370	15.85 (5.67)	280	16.29 (6.07)
MAAS	621	3.78 (0.83)	355	4.05 (0.75)	345	4.20 (0.84)	270	4.22 (0.86)

PSS-10 = Perceived Stress Scale (Cohen et al., 1983), score range 0–40; MAAS = Mindful Attention Awareness Scale (Brown and Ryan, 2003), score range 1–6: t0 = 0 weeks (baseline), t1 = 4 weeks (mid-intervention), t2 = 8 weeks (completion), t3 = 16 weeks (follow-up).

### 3.2 Outcome measures

Table 3 also shows descriptive statistics of the outcome variables on all assessment times.

#### 3.2.1 Self-reported stress

**Figure 2:**
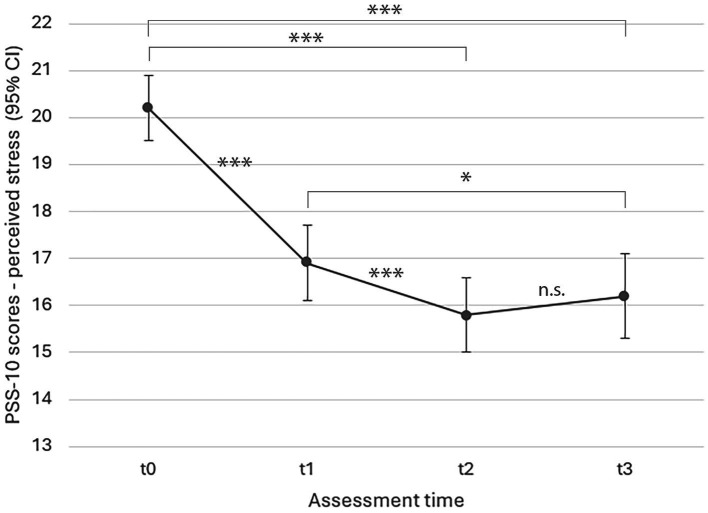
LMM model estimated marginal means for perceived stress over time (*p* < 0.001). PSS-10 = Perceived Stress Scale (Cohen et al., 1983); t0 = 0 weeks (baseline), t1 = 4 weeks (mid-intervention), t2 = 8 weeks (completion), t3 = 16 weeks (follow-up). All estimates (t1, t2, t3) were significant relative to t0 at *p* < 0.001; ***p < 0.001, **p < 0.01, *p < 0.05.

Model estimates for changes in self-reported stress over the course of the study, controlled for age, sex, and survey language, can be found in Figure 2. PSS-10 scores showed a significant decrease in self-reported stress in conjunction with the intervention (*F*(3, 902) = 123.969, *p* < 0.001; effect size *η_p_*^2^ = 0.28). As visible in Figure 2, stress scores decreased continuously from t0 to t2 and showed a non-significant small increase again at follow up. All estimates (t1, t2, t3) were significant relative to t0 at *p* < 0.001.

Mean frequencies and durations of self-practice per time lapse are provided in Table 4. Both had a significant effect on perceived stress as per ANOVA: PSS-10 scores were significantly lower if activity-based mindfulness exercises had been practiced on more days of the weeks preceding the assessment at t1 (*F*(3, 361) = 8.357, *p* < 0.001), t2 (*F*(3, 365) = 9.702, *p* < 0.001), and t3 (*F*(3, 275) = 4.651, *p =* 0.003), and likewise for longer self-practice durations in the weeks preceding t1 (*F*(4, 360) = 6.479, *p* < 0.001) and t2 assessment (*F*(4, 364) = 3.949, *p* = 0.004). The same held true for eurythmy exercises, with significantly lower stress scores in relation to more frequent self-practice during the weeks before t1 (*F*(3, 361) = 5.567, *p* < 0.001), t2 (*F*(3, 365) = 10.18, *p* < 0.001), and t3 assessment (*F*(3, 275) = 4.261, *p =* 0.006), and similarly for longer practice durations in the weeks prior to t1 (*F*(4, 360) = 5.297, *p <* 0.001) and t2 assessment (*F*(4, 364) = 3.03, *p* = 0.018).

**Table 4:** Self-practice times: mean frequency and duration.

	t1 *M* (SD)		t2 *M* (SD)		t3 *M* (SD)	
Exercises	Frequency	Duration	Frequency	Duration	Frequency	Duration
Activity-based	2.31 (0.89)	2.58 (0.88)	2.24 (0.89)	2.51 (0.82)	1.78 (0.83)	1.93 (0.89)
Eurythmy-based	2.34 (0.99)	2.68 (0.85)	2.30 (0.96)	2.58 (0.83)	1.84 (0.93)	2.10 (0.94)

Frequency (in days): 1 = 0–1, 2 = 2–3, 3 = 4–5, 4 = 6–7; duration (in minutes): 1 = 0, 2 = 1–10, 3 = 11–20, 4 = 21–30, 5 = more than 30; t0 = 0 weeks (baseline), t1 = 4 weeks (mid-intervention), t2 = 8 weeks (completion), t3 = 16 weeks (follow-up).

#### 3.2.2 Mindfulness

**Figure 3:**
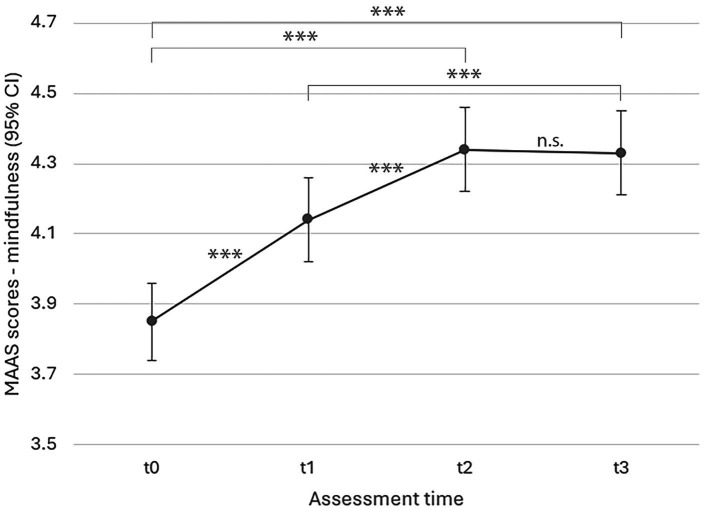
LMM model estimated marginal means for mindfulness over time (*p < 0.*001). MAAS = Mindful Attention Awareness Scale (Brown and Ryan, 2003); t0 = 0 weeks (baseline), t1 = 4 weeks (mid-intervention), t2 = 8 weeks (completion), t3 = 16 weeks (follow-up). All estimates (t1, t2, t3) were significant relative to t0 at *p* < 0.001; ***p < 0.001, **p < 0.01, *p < 0.05.

Figure 3 shows model estimates for changes in mindfulness over the course of the study, again controlled for age, sex, and survey language. There was a significant increase in mindfulness in conjunction with the intervention (*F*(3, 871) = 82.530, *p* < 0.001; effect size *η_p_*^2^ = 0.22), with scores steadily increasing from t0 to t3. All estimates (t1, t2, t3) were significant relative to t0 at *p* < 0.001.

One-way ANOVA yielded significant effects of frequency and duration of self-practice on mindfulness for activity-based mindfulness exercises, pointing to a significant increase in MAAS scores for participants that had practiced more frequently during the weeks preceding t2 (*F*(3, 341) = 7.629, *p <* 0.001) and t3 (*F*(3, 265) = 3.952, *p* = 0.009), and for longer durations in the weeks preceding t2 (*F*(4, 340) = 4.395, *p* = 0.002). Similarly, more frequent self-practice of eurythmy exercises was followed by higher MAAS scores at t2 (*F*(3, 341) = 10.600, *p* < 0.001), and t3 (*F*(3, 265) = 5.033, *p* = 0.002), as were longer self-practice durations in the weeks prior to t2 (*F*(4, 340) = 5.35, *p* < 0.001) and t3 assessment (*F*(4, 265) = 2.883, *p* = 0.023).

## 4 Discussion

The current study assessed an eight-week-long online MBI incorporating mindfulness-related practices from AM, using an observational repeated measures design and large-scale sample of healthy adults (*N* = 830). In line with our expectation, self-reported stress decreased significantly over the course of the intervention, with the most pronounced improvement occurring between baseline and week four, and the lower stress level maintained until 8 weeks post program completion. The reduction in stress is in line with research on the MBSR and other MBIs addressing stress in healthy adults (Khoury et al., 2015; Chiesa and Serretti, 2009). Furthermore, also in line with our expectation, our results showed a significant increase in mindfulness in conjunction with the intervention, MAAS scores increasing steadily from baseline through to the end of the intervention, with a small but non-significant further increase at follow up 8 weeks later. As such, our findings confirm that, akin to classical MBIs, the intervention indeed targets and cultivates mindfulness (Lamothe et al., 2016; Lampe and Müller-Hilke, 2021; Nyklíček and Kuijpers, 2008), albeit with different means.

We found large effect sizes for both increases in mindfulness and stress (Norouzian and Plonsky, 2018; Richardson, 2011). Studies using the PSS-10 and MAAS to evaluate MBSR showed comparable improvements in degree and effect size (Shapiro et al., 2005; Shapiro et al., 2011; Juul et al., 2018; Jensen et al., 2023; Birnie et al., 2010), although the interpretability of LMM effect sizes across studies is still being debated (Norouzian and Plonsky, 2018, Richardson, 2011).

We found improvements in stress and mindfulness to be maintained after 8 weeks, while MBI studies assessing longer follow-up intervals found positive effects to persist after 1 and even 3 years (Galante et al., 2021b; Beblo et al., 2024). In the current work a slight increase in stress scores was evident at follow up, which was however not significant, but could indicate that some degree of continued self-practice could be recommendable to sustain beneficial effects in the longer term. Indeed, according to our findings, frequency and duration of self-practice significantly impacted the outcomes, with more frequent and longer self-practice generally associated with larger beneficial changes in stress and mindfulness outcomes. This is consistent with findings from other MBIs, in which the extent of home practice was positively correlated with intervention outcomes (Parsons et al., 2017), but data on practice times are rarely reported in MBI studies (Jacobsen et al., 2022). A recent review reported only seven studies that assessed practice times, of which four found longer durations to lead to larger improvements in clinical outcomes (Lloyd et al., 2018).

This study had several limitations, including the observational single-arm design, which is however the norm for initial phases of feasibility testing of an intervention (Bowen et al., 2009). Future research should assess outcomes of the ABSR program using a randomized-controlled design and include longer follow-up intervals (e.g., 3, 6, 12, and 36 months). The survey completion rate in our study showed a rather high decrease across time, which is however a common finding of online studies with voluntary, anonymous, and uncompensated participation (Rostaminezhad et al., 2013; Bawa, 2016; Fish et al., 2016). Thus, despite the many advantages of online research, the non-committing format and perhaps also the technical demands may have presented a barrier impacting response rates (Gravesande et al., 2023). Further, due to resource constraints we were able to provide only 6 survey languages although the intervention was held in 9 languages, which may have contributed to a lowered response rate. The majority of non-completers left the study in the initial stages (after the first assessment), which is a typical pattern for online interventions in general and also in the context of MBSR studies specifically (De Paepe et al., 2018; Dobkin et al., 2012). Future studies should incorporate strategies to improve completion rate, which may include offering incentives for participation, as well as sending personally tailored email reminders to increase adherence over time (Meyerowitz-Katz et al., 2020), which would however require a non-anonymous study design.

This work had several strengths. It provides first indications for feasibility and beneficial outcomes regarding the online implementation of a novel MBI variant based on concepts and practices from AM, a well-established integrative medicine frame, thereby increasing plurality and diversity of options in the emerging field of MBIs. The current work demonstrated the intervention’s adaptability to an online format, which has the advantage of broader accessibility and affordability, as is the case for other MBIs delivered online (Spijkerman et al., 2016; Jayawardene et al., 2017; Sommers-Spijkerman et al., 2021; Mrazek et al., 2019; Gravesande et al., 2023; Teo et al., 2024).

Future studies should examine if this alternative MBI could be particularly supportive for certain subgroups of individuals. Although benefits of MBIs have been extensively documented (Davis and Hayes, 2011; Khoury et al., 2013; Enkema et al., 2020, Baer, 2003), not all types of mindfulness practices seem to be equally well suited for all types of people and purposes (Sedlmeier, 2023). Dobkin et al. (2012) for instance reviewed the literature for reasons for attrition, contraindications, and adverse events in classical MBIs, pointing out that individuals with severe chronic pain tend to be less likely to complete the program. Furthermore, they concluded that classical sitting meditation demands special care in the context of certain predispositions and psychopathologies, such as post-traumatic stress disorder, and is considered contraindicated for individuals with psychotic disorders (Dobkin et al., 2012). Indeed, also meta-analytic evidence suggested a lesser benefit of MBIs involving sitting meditation for individuals with pronounced fear symptoms (de Abreu Costa et al., 2019). Furthermore, specific age groups (Petersen and la Cour, 2016; Sedlmeier, 2023), as well as certain personality features (e.g., neuroticism, narcissism), appear to interact with the kind of mindfulness practice that is preferred or more beneficial for an individual (Sedlmeier, 2023; Tang and Braver, 2020). Although research in this context is only beginning to emerge (Dobkin et al., 2012), the advantage of being able to offer a plurality and diversity of mindfulness approaches and practices is evident given the distinctive needs and corresponding fit, or lack thereof. Further research examining a broader range of outcomes and clinical populations will be necessary to determine for which groups of people this specific mindfulness approach based on AM would be especially suitable. For example, it is conceivable that the activity-based exercises of the ABSR could be supportive for individuals for whom sitting quietly while focusing attention on their inner world is associated with high levels of anxiety or impossible for other reasons. Importantly, future research should consider to assess mental health status and diagnoses of participants to find out who demonstrates most benefits, and conversely, if there are individuals for whom the intervention is less suitable or contraindicated.

## 5 Conclusion

While the current research provides promising preliminary indications regarding the online implementation of this novel MBI based on practices from AM, these findings need to be confirmed in randomized-controlled studies given the limitations of the current work, in particular its observational single-arm design and completion rate. Nonetheless, the study adds a unique contribution to existent MBIs, which is significant in view of the need for diverse approaches to meet the heterogeneity of individual predispositions and clinical needs. It remains to established by forthcoming research for which subgroups of individuals or clinical features this approach could be especially beneficial, or less suitable.

## Data Availability

The raw data supporting the conclusions of this article will be made available by the authors, without undue reservation.

## Footnotes

^1^ABSR International (Eurythmy4you).
